# Treated Incidence of Psychotic Disorders in the Multinational EU-GEI Study

**DOI:** 10.1001/jamapsychiatry.2017.3554

**Published:** 2017-12-06

**Authors:** Hannah E. Jongsma, Charlotte Gayer-Anderson, Antonio Lasalvia, Diego Quattrone, Alice Mulè, Andrei Szöke, Jean-Paul Selten, Caitlin Turner, Celso Arango, Ilaria Tarricone, Domenico Berardi, Andrea Tortelli, Pierre-Michel Llorca, Lieuwe de Haan, Julio Bobes, Miguel Bernardo, Julio Sanjuán, José Luis Santos, Manuel Arrojo, Cristina Marta Del-Ben, Paulo Rossi Menezes, Robin M. Murray, Bart P. Rutten, Peter B. Jones, Jim van Os, Craig Morgan, James B. Kirkbride

**Affiliations:** 1 Department of Psychiatry, University of Cambridge, Cambridge, England; 2 Department of Health Service and Population Research, Institute of Psychiatry, King’s College London, London, England; 3 Section of Psychiatry, Azienda Ospedaliera Universitaria Integrata di Verona, Verona, Italy; 4 Department of Psychosis Studies, Institute of Psychiatry, King’s College London, London, England; 5 Unit of Psychiatry, “P. Giaccone” General Hospital, Palermo, Italy; 6 Institut National de la Santé et de la Recherche Médicale, U955, Créteil, France; 7 Rivierduinen Institute for Mental Health Care, Leiden, the Netherlands; 8 Department of Psychiatry and Neuropsychology, School for Mental Health and Neuroscience, Maastricht University Medical Centre, Maastricht, the Netherlands; 9 Cambridge Institute of Public Health, University of Cambridge, Cambridge, England; 10 Department of Child and Adolescent Psychiatry, Hospital General Universitario Gregorio Marañón, School of Medicine, Universidad Complutense, Investigación Sanitaria del Hospital Gregorio Marañón, Madrid, Spain.; 11 Centro de Investigación Biomédica en Red de Salud Mental, Madrid, Spain.; 12 Department of Medical and Surgical Sciences, Psychiatry Unit, Alma Mater Studiorum Università di Bologna, Bologna, Italy; 13 Etablissement Public de Santé Maison Blanche, Paris, France; 14 EA 7280 Npsydo, Université Clermont Auvergne, Clermont-Ferrand, France; 15 Department of Psychiatry, Early Psychosis Section, Academic Medical Centre, University of Amsterdam, Amsterdam, the Netherlands; 16 Department of Medicine, Psychiatry Area, School of Medicine, Universidad de Oviedo, Centro de Investigación Biomédica en Red de Salud Mental, Oviedo, Spain; 17 Barcelona Clinic Schizophrenia Unit, Neuroscience Institute, Hospital Clinic, Department of Medicine, University of Barcelona, Institut d’Investigacions Biomèdiques August Pi i Sunyer, Centro de Investigación Biomédica en Red de Salud Mental, Barcelona, Spain.; 18 Department of Psychiatry, School of Medicine, Universidad de Valencia, Centro de Investigación Biomédica en Red de Salud Mental, Valencia, Spain; 19 Department of Psychiatry, Servicio de Psiquiatría Hospital “Virgen de la Luz,” Cuenca, Spain; 20 Department of Psychiatry, Psychiatric Genetic Group, Instituto de Investigación Sanitaria de Santiago de Compostela, Complejo Hospitalario Universitario de Santiago de Compostela, Spain; 21 Division of Psychiatry, Department of Neuroscience and Behaviour, Ribeirão Preto Medical School, University of São Paulo, São Paulo, Brazil; 22 Department of Preventive Medicine, Faculdade de Medicina, Universidade of São Paulo, São Paulo, Brazil; 23 CAMEO Early Intervention Service, Cambridgeshire and Peterborough National Health Service Foundation Trust, Cambridge, England; 24 Department Psychiatry, Brain Center Rudolf Magnus, Utrecht University Medical Centre, Utrecht, the Netherlands; 25 Psylife Group, Division of Psychiatry, University College London, London, England

## Abstract

**Importance:**

Psychotic disorders contribute significantly to the global disease burden, yet the latest international incidence study of psychotic disorders was conducted in the 1980s.

**Objectives:**

To estimate the incidence of psychotic disorders using comparable methods across 17 catchment areas in 6 countries and to examine the variance between catchment areas by putative environmental risk factors.

**Design, Setting, and Participants:**

An international multisite incidence study (the European Network of National Schizophrenia Networks Studying Gene-Environment Interactions) was conducted from May 1, 2010, to April 1, 2015, among 2774 individuals from England (2 catchment areas), France (3 catchment areas), Italy (3 catchment areas), the Netherlands (2 catchment areas), Spain (6 catchment areas), and Brazil (1 catchment area) with a first episode of nonorganic psychotic disorders (*International Statistical Classification of Diseases and Related Health Problems, Tenth Revision* [*ICD-10*] codes F20-F33) confirmed by the Operational Criteria Checklist. Denominator populations were estimated using official national statistics.

**Exposures:**

Age, sex, and racial/ethnic minority status were treated as a priori confounders. Latitude, population density, percentage unemployment, owner-occupied housing, and single-person households were treated as catchment area–level exposures.

**Main Outcomes and Measures:**

Incidence of nonorganic psychotic disorders (*ICD-10* codes F20-F33), nonaffective psychoses (*ICD-10* codes F20-F29), and affective psychoses (*ICD-10* codes F30-F33) confirmed by the Operational Criteria Checklist.

**Results:**

A total of 2774 patients (1196 women and 1578 men; median age, 30.5 years [interquartile range, 23.0-41.0 years]) with incident cases of psychotic disorders were identified during 12.9 million person-years at risk (crude incidence, 21.4 per 100 000 person-years; 95% CI, 19.4-23.4 per 100 000 person-years). A total of 2183 patients (78.7%) had nonaffective psychotic disorders. After direct standardization for age, sex, and racial/ethnic minority status, an 8-fold variation was seen in the incidence of all psychotic disorders, from 6.0 (95% CI, 3.5-8.6) per 100 000 person-years in Santiago, Spain, to 46.1 (95% CI, 37.3-55.0) per 100 000 person-years in Paris, France. Rates were elevated in racial/ethnic minority groups (incidence rate ratio, 1.6; 95% CI, 1.5-1.7), were highest for men 18 to 24 years of age, and were lower in catchment areas with more owner-occupied homes (incidence rate ratio, 0.8; 95% CI, 0.7-0.8). Similar patterns were observed for nonaffective psychoses; a lower incidence of affective psychoses was associated with higher area-level unemployment (incidence rate ratio, 0.3; 95% CI, 0.2-0.5).

**Conclusions and Relevance:**

This study confirmed marked heterogeneity in risk for psychotic disorders by person and place, including higher rates in younger men, racial/ethnic minorities, and areas characterized by a lower percentage of owner-occupied houses.

## Key Points

**Question:**

Does the treated incidence of psychotic disorders vary across 17 settings in 6 countries, and to what extent is this explained by individual and catchment area–level risk factors?

**Findings:**

The overall crude incidence of psychotic disorders in this international multisite incidence study was 21.4 per 100 000 person-years, and after standardization this incidence varied 8-fold between catchment areas. Higher incidence was also associated with younger age, male sex, racial/ethnic minority status, and lower catchment area–level owner-occupancy; similar variation was found independently for nonaffective and affective psychoses.

**Meaning:**

The incidence of psychotic disorders varied by person and place, indicating that both individual and catchment area–level risk factors are important in predicting incidence.

## Teaser

This international multisite incidence study estimates the incidence of psychotic disorders across 17 catchment areas in 6 countries and examines the variance between catchment areas by putative environmental risk factors.

## Introduction

The World Health Organization Ten-Country Study,^1^ the most recent multicenter international study of the incidence of psychotic disorders, was widely interpreted as demonstrating worldwide homogeneity in rates of schizophrenia and other psychotic disorders. In fact, there was a 2.5-fold variation in broadly defined nonaffective psychoses.

Subsequent studies^2^ showed that psychotic disorders vary considerably across replicable social and environmental gradients, including increased rates among men, younger adults,^5^ racial/ethnic minority groups,^4^ and with urban birth and upbringing.^6^ This finding built on earlier epidemiologic studies from the United States^7^ and Europe,^10^ which revealed strong associations between neighborhood social deprivation and greater rates of psychosis. Nevertheless, to our knowledge, there has been no international comparison of the incidence of psychotic disorders since the World Health Organization study.^1^ We estimated the incidence of psychotic disorders across 17 catchment areas in 6 countries using comparable methods as part of the European Network of National Schizophrenia Networks Studying Gene-Environment Interactions (EU-GEI) study. Specifically, we tested whether differences in incidence could be attributed to putative social and environmental factors, including individual age, sex, and race/ethnic minority status, catchment area–level latitude,^13^ population density,^6^ unemployment, and proportion of single-person households and owner-occupied homes as markers of social disadvantage.

## Methods

### Study Design and Settings

The EU-GEI study is a multicenter incidence and case-sibling-control study of genetic and environmental determinants of psychotic disorders. Centers for the incidence study were England (n = 2; southeast London, Cambridgeshire and Peterborough), France (n = 3; 20th arrondissement of Paris, Val-de-Marne, Puy-de-Dôme), the Netherlands (n = 2; central Amsterdam, Gouda and Voorhout), Italy (n = 3; part of the Veneto region, Bologna municipality, and the city of Palermo), Spain (n = 6; Madrid [Vallecas], Barcelona, Valencia, Oviedo, Santiago, and Cuenca), and Brazil (n = 1; Ribeirão Preto). Catchment areas ranged from rural (Cuenca, 11 people per square kilometer) to urban (Paris, 33 260 people per square kilometer). Written informed consent was obtained from those who agreed to participate in the case-control study; otherwise, ethical approval was obtained to extract basic demographic and clinical details from patient records from local research ethics committees in each catchment area: South London and Maudsley and Institute of Psychiatry Research Ethics Committee; National Research Ethics Service Committee East of England–East Cambridge; Medisch-Ethische Toetsingscommissie van het Academisch Centrum te Amsterdam; Comité Ético de Investigación Clínica Hospital Gregorio Marañón; Comité Ético de Investigación Clínica del Hospital Clinic de Barcelona; Comité Ético de Investigación Clínica del Hospital Clinic Universitari de Valencia; Comité Ética de la Investigación Clínica del Principado de Asturias; Comité Ético de Investigación Clínica de Galicia; Comité Ético de Investigación Clínica del Hospital Virgen de la Luz de Cuenca; Comité de Protéction des Personnes–CPP Île de France IX; Comitato Etico Policlinico S Orsola Malpighi; Comitato Etico Azienda Ospedaleria Universitaria di Verona; Comitato Etico Palermo 1, Azienda Ospedaliera Policlinico “Paolo Giaccone”; and Research Ethics Committee of the clinical Hospital of Ribeirão Preto Medical School, University of São Paulo, Brazil.

### Participants

We identified all individuals who contacted mental health services in our catchment areas for a suspected first episode of psychosis (FEP). Case ascertainment varied from 12 months (London, England) to 48 months (Val-de-Marne, France) (Table 1), with a median of 25 months (interquartile range [IQR], 24-36 months). Case ascertainment predominantly took place between May 1, 2010, and April 1, 2015 (eTable 1 in the Supplement), and involved trained researchers making regular contact with all secondary and tertiary mental health care professionals to identify potential cases. In all countries, it was uncommon for people to be treated for FEP in primary care; instead, a patient with a suspicion of psychosis would typically be referred to specialist mental health services. Research teams were overseen by a psychiatrist with experience in epidemiologic research and included trained research nurses and clinical psychologists. Teams received training in epidemiologic principles and incidence study design to minimize nondifferential ascertainment bias across different local and national health care systems.

**Table 1.** Population and Sample Characteristics by Catchment Area

Catchment Area	Case Ascertainment, mo	Total Person-years	Men, No. (%)	Racial/Ethnic Majority, No. (%)	Total Cases, No.	Nonaffective Psychoses, No. (%)	Affective Psychoses, No. (%)	Men, No. (%)	Racial/Ethnic Majority, No. (%)	Median Age at First Contact, y (IQR)
England										
Southeast London	12	426 453	212 981 (49.9)	175 706 (41.2)	262	245 (93.5)	17 (6.5)	141 (53.8)	64 (24.4)	32.0 (24.0-43.0)
Cambridgeshire	36	1 554 423	782 607 (50.4)	1 238 172 (79.7)	266	185 (69.6)	77 (29.0)	151 (56.7)	164 (61.7)	28.0 (22.0-37.0)
The Netherlands										
Amsterdam	36	621 141	313 287 (50.4)	293 709 (47.3)	292	264 (90.4)	27 (9.3)	188 (64.4)	89 (30.5)	31.0 (24.0-42.5)
Gouda and Voorhout	36	766 770	384 975 (50.2)	651 786 (85.0)	167	122 (73.5)	39 (23.4)	101 (60.8)	127 (76.2)	29.0 (22.0-38.0)
Spain										
Madrid	22	414 786	205 367 (49.5)	329 425 (79.4)	89	72 (80.9)	12 (13.5)	58 (63.8)	76 (86.4)	30.0 (23.0-40.0)
Barcelona	25	883 894	426 258 (48.2)	688 283 (77.9)	108	96 (88.9)	8 (7.4)	62 (57.4)	82 (75.9)	28.0 (21.5-35.5)
Valencia	24	364 192	180 698 (49.6)	299 983 (82.4)	58	51 (87.9)	5 (8.6)	32 (55.1)	48 (82.7)	28.0 (24.0-39.0)
Oviedo	25	462 624	226 890 (49.1)	428 483 (92.6)	82	66 (80.5)	12 (14.6)	40 (48.8)	67 (81.7)	32.0 (24.0-43.0)
Santiago	25	574 944	286 767 (49.9)	556 192 (96.7)	36	30 (83.3)	5 (13.9)	20 (55.6)	35 (97.2)	33.0 (25.0-43.5)
Cuenca	23	195 074	102 697 (52.6)	160 724 (82.4)	27	26 (96.3)	0 (0.0)	21 (77.8)	20 (74.1)	26.0 (21.0-37.0)
France										
Paris	24	268 362	128 162 (47.8)	179 220 (66.8)	120	108 (90.0)	12 (10.0)	83 (69.2)	66 (55.0)	30.5 (22.5-40.5)
Val-de-Marne	48	510 632	242 334 (47.5)	342 091 (77.0)	212	134 (63.2)	76 (35.9)	107 (51.2)	142 (67.9)	30.0 (23.0-42.0)
Puy-de-Dôme	24	226 545	113 579 (50.1)	213 784 (94.4)	42	28 (66.7)	14 (33.3)	28 (66.7)	NA	31.0 (22.0-46.0)
Italy										
Bologna	48	931 746	453 320 (48.9)	789 474 (85.1)	165	130 (78.8)	35 (21.2)	86 (52.1)	116 (70.3)	30.0 (23.0-41.0)
Veneto	36	505 508	259 282 (51.3)	446 523 (88.3)	104	82 (78.9)	14 (13.5)	56 (53.9)	83 (79.8)	35.5 (28.0-42.0)
Palermo	44	1 594 882	781 002 (49.0)	1 493 857 (93.7)	179	155 (86.6)	23 (12.9)	100 (55.9)	158 (88.3)	30.0 (24.0-40.0)
Brazil										
Ribeirão Preto	36	2 631 689	1 299 112 (49.4)	1 745 638 (66.3)	565	389 (68.9)	175 (31.0)	304 (53.8)	302 (53.5)	32.0 (25.0-43.0)
Total	NA	12 933 670	6 401 911 (49.5)	9 971 270 (77.1)	2,774	2183 (78.7)	551 (19.9)	1578 (57.0)	1639 (60.1)	30.5 (23.0-41.0)
χ^2^; *P* value	NA	NA	4.4 × 10^3^; <.001	1.4 × 10^6^; <.001	NA	172.6; <.001	189.9; <.001	34.3; .005	453.0; <.001	51.3; <.001

Abbreviations: IQR, interquartile range; NA, not applicable.

Potential participants with FEP were included if they met the following criteria: resident within the catchment area at first presentation; 18 to 64 years of age; and presentation with a clinical diagnosis for an untreated FEP, even if longstanding (*International Statistical Classification of Diseases and Related Health Problems, Tenth Revision* [*ICD-10*] codes F20-F33). We excluded individuals who had previous contact with mental health services for psychosis, evidence of psychotic symptoms precipitated by an organic cause, and transient psychotic symptoms resulting from acute intoxication, as defined by the *ICD-10* (codes F1X.5)

For participants who met these criteria, we obtained research-based diagnoses using the Operational Criteria Checklist algorithm (OPCRIT) to ensure comparability of diagnoses across catchment areas. The OPCRIT has high interrater reliability generally,^14^ and in our study after training (κ = 0.7). Assessment with OPCRIT was based on a semistructured clinical interview or review of case notes and other relevant information. The clinical interview schedule used at each site followed local expertise, including the Schedules for Clinical Assessment in Neuropsychiatry^15^ (United Kingdom and Italy), the Comprehensive Assessment of Symptoms and History^16^ (the Netherlands), the Structured Interview for *DSM-IV* (Brazil),^17^ and the Diagnostic Interview for Genetic Studies^18^ (France). Where OPCRIT assessment was not possible, we relied on clinical diagnoses.

### Population at Risk

We estimated the population at risk, those 18 to 64 years of age, in each catchment area from the most accurate local or national routine demographic data available (eTable 2 in the Supplement), stratified by age (18-24 years, then 5-year bands), sex, and racial/ethnic minority status. We multiplied the population by case ascertainment duration (in years) to estimate person-years at risk.

### Measures

Our primary outcome was an OPCRIT-confirmed *ICD-10* diagnosis of any clinically relevant psychotic disorder (*ICD-10* codes F20-F33). This broad phenotype was considered alongside 2 secondary outcomes: nonaffective psychoses (*ICD-10* codes F20-F29) and affective psychoses (*ICD-10* codes F30-F33).

Data on age group (as above), sex, racial/ethnic minority status, and country of birth were collected at baseline for all participants using the Medical Research Council Sociodemographic Questionnaire^19^ and case notes. We defined a binary variable to distinguish between the racial/ethnic majority population in each catchment area, and all other racial/ethnic minority groups. In each country, the racial/ethnic majority population was classified as the majority (white) racial/ethnic group, following national conventions (eAppendix 1 and eTable 2 in the Supplement), with all other groups classified as the racial/ethnic minority.

Latitude was estimated in degrees from the equator. Population density was derived as number of inhabitants per square kilometer, based on official total population estimates. We derived 3 measures of the social environment (unemployment, owner-occupied housing, and single-person households) from the 2011 European Household and Population Census,^20^ a decennial census that provides comparable data at a provincial level (NUTS-2 [Nomenclature of Territorial Units for Statistics–2] regions). Equivalent data for Ribeirão Preto were derived from the 2010 National Census of Brazil.^21^ Duration of untreated psychosis (in weeks) was estimated for descriptive purposes, assessed via the Nottingham Onset Schedule,^22^ and based on time from onset of symptoms to first contact with secondary mental health services for suspected psychosis. For deviations from the protocol, see eAppendix 1 in the Supplement.

### Missing Data

Seven of 2774 cases (0.3%) were missing data on age or sex, and were excluded from direct standardization and statistical modeling, but retained for crude incidence rate estimation. Except for Puy-de-Dôme, France (eAppendix 1 in the Supplement), we coded any participants missing data on racial/ethnic minority status (n = 5 [0.2%]) to the racial/ethnic majority group.

### Statistical Analysis

For each outcome, we estimated crude incidence rates per 100 000 person-years and 95% CIs by catchment area and sociodemographic characteristics. Next, we used direct standardization for age-band and sex and for age-band, sex, and racial/ethnic minority status to investigate variation in rates between catchment areas. We used the total population of England and Wales (2011 Census^23^) as our standard population, and estimated standardized incidence ratios using the overall sample incidence rate as the reference category. Finally, we used random-effects (intercepts) Poisson regression to investigate variance in incidence by sociodemographic and environmental factors, accounting for the hierarchical structure of the data set. Age, sex, their interaction, and racial/ethnic minority status were treated as a priori confounders. We entered catchment area–level variables into our models one at a time based on the strength of association with incidence in univariable analyses, assessed via Akaike Information Criterion (lower scores indicate better model fit). Model building was assessed via likelihood ratio test. Analyses were carried out in Stata, version 13 (StataCorp). Results for secondary outcomes, as well as sensitivity analyses, are reported in eAppendix 2 in the Supplement. *P* < .05 (2-sided) was considered significant.

## Results

### Participant Characteristics

We identified 2774 people presenting with a first episode of psychotic disorder, as defined by *ICD-10* criteria, during 12.94 million person-years, corresponding to a crude incidence of 21.4 (95% CI, 19.4-23.4) per 100 000 person-years. A total of 1578 participants with FEP were men (56.9%), varying from 48.8% (40 of 82; Oviedo, Spain) to 77.8% (21 of 27; Cuenca, Spain) (χ^2^ = 34.3; *P* = .005; Table 1). A total of 1091 participants with FEP were from a racial/ethnic minority background (39.8%), varying from 2.8% (1 of 36; Santiago, Spain) to 75.6% (198 of 262; Southeast London, England) (χ^2^ = 455.8; *P* < .001). By comparison, almost 49.5% of the population at risk were men (6 401 911 of 12 933 665), and 22.9% were from a racial/ethnic minority group (2 962 395 of 12 933 665).

Median age at first contact was 30.5 years (IQR, 23.0-41.0 years), varying from 26.0 years (IQR, 21.0-37.0 years) in Cuenca, Spain, to 35.5 years (IQR, 28.0-42.0 years) in Veneto, Italy (Kruskal-Wallis χ^2^_16_ = 51.3; *P* < .001). First contact was earlier in men (28.0 years; IQR, 22.0-38.0 years) than in women (34.0 years; IQR, 26.0-45.0 years; Mann-Whitney test = –11.1; *P* < .001), but did not differ by racial/ethnic minority status (Mann-Whitney test = 1.0; *P* = .31). Median duration of untreated psychosis was 8.0 weeks (IQR, 2.0-35.0 weeks), varying from 2.5 weeks (IQR, 1.0-7.0 weeks) in Madrid, Spain, to 26.0 weeks (IQR, 2.0-77.0 weeks) in Cuenca, Spain (eTable 1 in the Supplement; Kruskal-Wallis χ^2^_15_ = 119.7; *P* < .001).

### Variation in the Incidence of FEP

**Figure 1.**
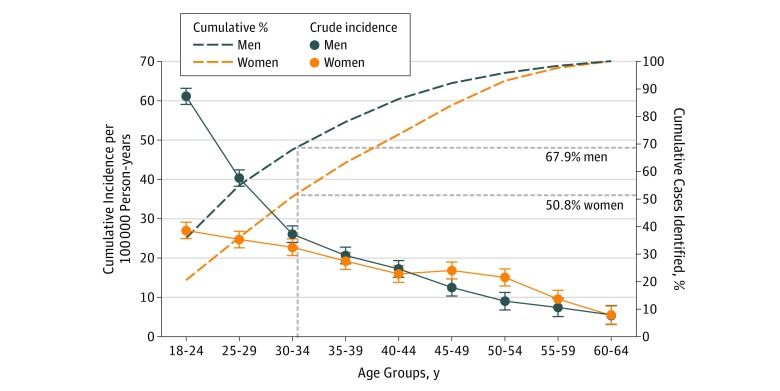
Crude Incidence and Cumulative Percentage of Psychotic Disorders, by Age and Sex A total of 1070 of 1577 men (67.9%) and 605 of 1190 women (50.8%) presented to mental health services before 35 years of age. Error bars indicate 95% CIs.

The age pattern of the incidence of FEP differed between men and women (Figure 1; likelihood ratio test χ^2^_8_ = 119.3; *P* < .001). Crude rates of FEP peaked for men between 18 and 24 years of age (61.0 per 100 000 person-years; 95% CI, 59.0-63.1 per 100 000 person-years) and decreased steeply thereafter. For women, the incidence of FEP also peaked in the youngest age group (18-24 years) at 27.0 per 100 000 person-years (95% CI, 24.9-29.1 per 100 000 person-years), but decreased more gradually thereafter, with a small secondary peak between 50 and 54 years of age. Rates were higher in racial/ethnic minority groups (incidence rate ratio [IRR], 1.59; 95% CI, 1.46-1.72) after multivariable adjustment for age, sex, their interaction, and relevant catchment area–level characteristics.

**Figure 2.**
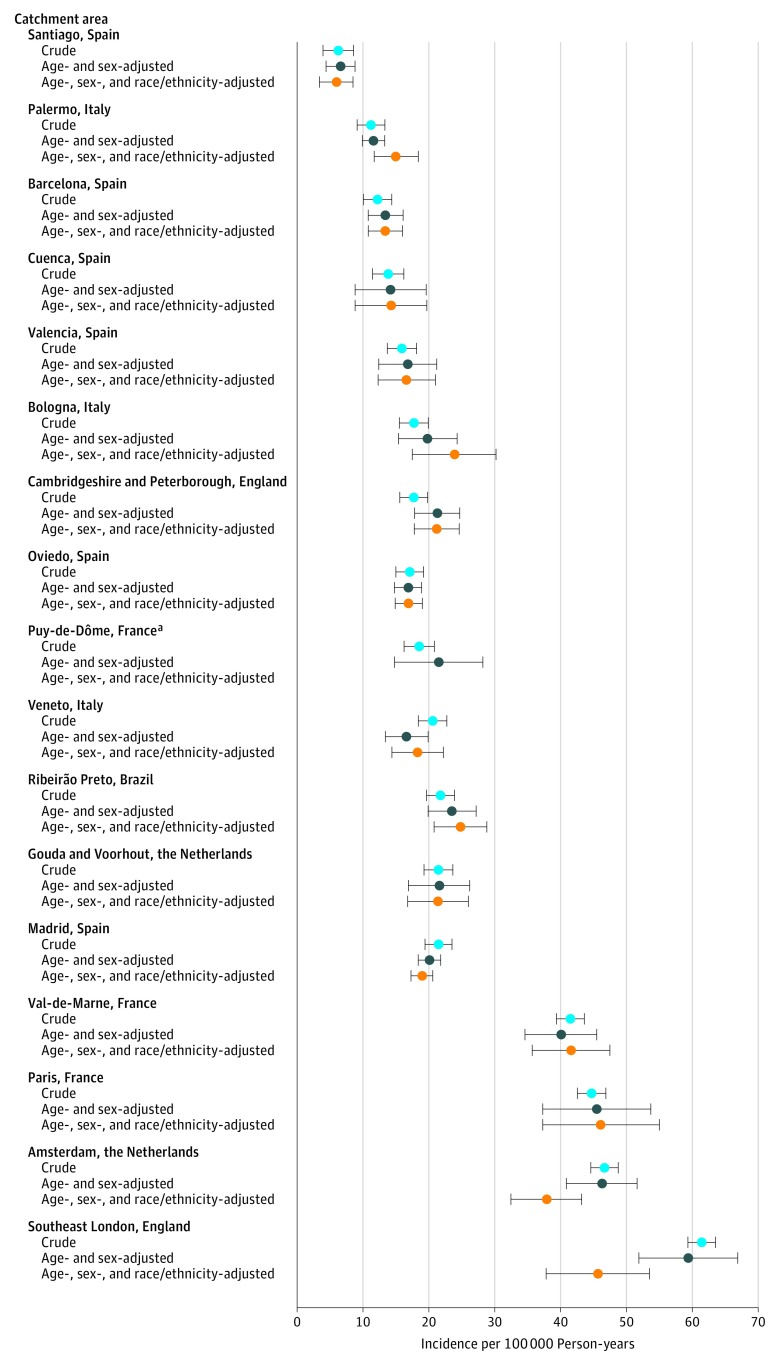
Crude Age- and Sex-Standardized and Age-, Sex-, and Race/Ethnicity-Standardized Incidence Rates per Catchment Area Crude incidence rates vary 10-fold between catchment areas, and age-, sex-, and race/ethnicity-adjusted rates vary 8-fold. Error bars indicate 95% CIs. ^a^Data by race/ethnicity were not available.

We observed 10-fold variation in crude incidence of FEP across our catchment areas (Figure 2 and Table 2), from 6.3 (95% CI, 3.9-8.6) per 100 000 person-years in Santiago, Spain, to 61.4 (95% CI, 59.4-63.5) per 100 000 person-years in southeast London, England. Age-sex standardization had a negligible effect on this variation (Figure 2). Additional standardization for racial/ethnic minority status attenuated variance, although an almost 8-fold variation remained; standardized incidence ratios varied from 0.29 (95% CI, 0.21-0.40) in Santiago, Spain, to 2.21 (95% CI, 1.84-2.65) in Paris, France.

**Table 2.** Crude Incidence Rates and Direct ASM-Standardized Incidence Ratios of All FEPs, Nonaffective Psychoses, and Affective Psychoses by Catchment Area

Catchment Area	All Psychotic Disorders		Nonaffective Psychoses		Affective Psychoses	
	Crude Incidence Rate (95% CI)	ASM-Standardized Ratio (95% CI)^a^	Crude Incidence Rate (95% CI)	ASM-Standardized Ratio (95% CI)^a^	Crude Incidence Rate (95% CI)	ASM-Standardized Ratio (95% CI)^a^
England						
Southeast London	61.4 (59.4-63.5)	2.19 (1.93-2.48)	57.5 (50.7-65.1)	2.50 (2.19-2.85)	4.0 (2.5-6.4)	1.07 (0.66-1.74)
Cambridgeshire	17.1 (15.0-19.2)	0.81 (0.71-0.92)	11.9 (10.3-13.7)	0.71 (0.61-0.82)	5.0 (4.0-6.2)	1.19 (0.94-1.51)
The Netherlands						
Amsterdam	46.7 (44.6-48.7)	1.81 (1.61-2.05)	42.2 (37.5-47.8)	2.03 (1.79-2.31)	4.3 (3.0-6.3)	1.00 (0.68-1.47)
Gouda and Voorhout	21.8 (19.7-23.9)	1.19 (1.01-1.39)	15.9 (13.3-19.0)	1.13 (0.94-1.36)	5.1 (3.7-7.0)	1.29 (0.93-1.78)
Spain						
Madrid	21.5 (19.3-23.6)	1.02 (0.83-1.26)	17.4 (13.8-21.9)	1.08 (0.86-1.37)	2.9 (1.6-5.1)	0.60 (0.34-1.05)
Barcelona	12.2 (10.1-14.4)	0.64 (0.53-0.78)	10.9 (8.9-13.3)	0.73 (0.59-0.89)	0.9 (0.5-1.8)	0.21 (0.11-0.43)
Valencia	15.9 (13.7-18.2)	0.79 (0.61-1.03)	14.0 (10.6-18.4)	0.88 (0.67-1.17)	1.4 (0.6-3.3)	0.36 (0.15-0.86)
Oviedo	17.7 (15.5-19.9)	1.14 (0.92-1.42)	14.3 (11.2-18.2)	1.15 (0.90-1.47)	2.6 (1.5-4.6)	0.83 (0.47-1.48)
Santiago	6.3 (3.9-8.6)	0.29 (0.21-0.40)	5.2 (3.6-7.5)	0.30 (0.21-0.43)	0.9 (0.4-2.1)	0.19 (0.08-0.46)
Cuenca	13.8 (11.5-16.2)	0.68 (0.47-1.00)	13.3 (9.1-19.6)	0.83 (0.56-1.22)	NA	NA
France						
Paris	44.7 (42.6-46.9)	2.21 (1.84-2.65)	40.2 (33.3-48.6)	2.45 (2.02-2.97)	4.5 (2.5-7.9)	1.38 (0.78-2.45)
Val-de-Marne	41.5 (39.4-43.6)	1.99 (1.73-2.29)	26.2 (22.2-31.1)	1.63 (1.37-1.94)	14.9 (11.9-18.6)	3.50 (2.75-4.45)
Puy-de-Dôme	18.5 (16.3-20.8)	NA	12.4 (8.5-17.9)	NA	6.2 (3.7-10.4)	NA
Italy						
Bologna	17.7 (15.6-19.8)	1.01 (0.87-1.19)	14.0 (11.7-16.6)	1.02 (0.85-1.22)	3.8 (2.7-5.2)	1.05 (0.74-1.47)
Veneto	20.6 (18.4-22.7)	0.88 (0.72-1.06)	16.2 (13.1-20.1)	0.87 (0.70-1.09)	2.8 (1.6-4.7)	0.60 (0.35-1.01)
Palermo	11.2 (9.1-13.3)	0.72 (0.62-0.83)	9.7 (8.3-11.4)	0.81 (0.69-0.96)	1.4 (1.0-2.2)	0.38 (0.25-0.58)
Brazil						
Ribeirão Preto	21.5 (19.4-23.5)	0.91 (0.83-1.00)	14.8 (13.4-16.3)	0.81 (0.72-0.90)	6.6 (5.7-7.7)	1.36 (1.14-1.61)
Total	21.4 (19.4-23.4)	1 [Reference]	16.9 (16.2-17.6)	1 [Reference]	4.3 (3.9-4.6)	1 [Reference]

Abbreviations: ASM, age, sex, and racial/ethnic minority; FEP, first episode of psychosis; NA, not applicable.

^a^ ASM directly standardized rates to the 2011 population structure of England and Wales.

The association between crude incidence of FEP and catchment area–level exposures are shown in the eFigure in the Supplement; univariable random intercepts Poisson regression showed that greater owner-occupancy (IRR for a 10% increase, 0.73; 95% CI, 0.65-0.81) and unemployment (IRR for a 10% increase, 0.54; 95% CI, 0.34-0.84) were associated with a lower incidence of FEP, while percentage of single-person households (IRR for a 10% increase, 1.68; 95% CI, 1.24-2.27) was associated with a higher incidence of FEP (Table 3). A null random intercepts Poisson model confirmed substantial variation in incidence by catchment area (σ = 0.32; *P* = .006), which persisted after adjustment for age, sex, their interaction, and racial/ethnic minority status (σ = 0.23; *P* = .007). In multivariable analyses, incidence of FEP was 1.59 (95% CI, 1.46-1.72) times higher in racial/ethnic minority groups compared with the racial/ethnic majority population, and lower in catchment areas with more owner-occupied homes (IRR for a 10% increase in owner-occupancy, 0.76; 95% CI, 0.70-0.83). No other setting-level variables, including latitude (IRR, 0.99; 95% CI 0.97-1.01), improved our final model (Table 3), where residual variance by catchment area remained, albeit attenuated (σ = 0.06; *P* = .02). Similar results were observed for nonaffective and affective psychoses separately (eAppendix 2 in the Supplement).

**Table 3.** Univariable and Multivariable Random Intercepts Poisson Regression of All FEPs

Variable	Univariable IRR (95% CI)	Univariable Wald *P* Value	Multivariable IRR (95% CI)^a^	Multivariable LRT *P* Value
Individual level				
Minority status (vs majority)	1.69 (1.56-1.84)	<.001	1.59 (1.46-1.72)	<.001
Setting level				
Distance from equator (degrees)	1.03 (1.00-1.07)	.07	0.99 (0.97-1.01)	.46
Population density (per 1000 people per km^2^)	1.02 (0.99-1.05)	.15	1.01 (0.99-1.02)	.44
** Owner-occupancy (10%)**	0.73 (0.65-0.81)	<.001	0.76 (0.70-0.83)	<.001
Single-person households (10%)	1.68 (1.24-2.27)	.001	1.06 (0.78-1.43)	.73
Unemployment (10%)	0.54 (0.34-0.84)	.007	0.90 (0.66-1.23)	.51

Abbreviations: FEP, first episode of psychosis; IRR, Incidence rate ratio; LRT, likelihood ratio test.

^a^ Models adjusted for age, sex, their interaction, and, for setting-level variables, race/ethnicity. IRR for nonsignificant setting-level variables obtained from a model after additional adjustment for owner-occupancy.

## Discussion

### Principal Findings

We observed substantial variation in the incidence of FEP across 17 catchment areas in 6 countries, confirming differential risk by place and person. In line with previous studies, we observed higher rates of all psychotic disorders in racial/ethnic minority groups^4^ and among young people,^5^ particularly for men.^24^ We confirmed a small but robust secondary peak in the risk of all FEPs for women older than 45 years. Catchment areas with higher owner-occupancy levels were associated with lower incidence rates of FEPs, implicating socioeconomic factors in the presentation of psychotic disorders, in line with findings of previous research.^25^

### Comparison With the Previous Literature

Overall, our incidence rates were consistent with those in the literature, although between-study heterogeneity in methods, inclusion criteria, and diagnoses studied make direct comparisons difficult. For example, the incidence of broadly defined schizophrenia in the World Health Organization Ten-Country Study^1^ varied from 15 to 42 per 100 000 person-years, although that study used a different age range (15-54 years) and did not consider affective psychoses.^1^ In our study, comparable rates of nonaffective psychoses varied from 5 to 41 per 100 000 person-years after standardization for age, sex, and racial/ethnic minority status. A systematic review limited to England observed a pooled crude incidence of all psychotic disorders of 32.7 per 100 000 person-years,^3^ somewhat higher than the overall crude incidence rate we observed (21.4 per 100 000 person-years). Such comparisons should be interpreted with caution, given heterogeneity in estimation methods and setting; few incidence studies have been conducted in southern Europe until recently,^27^ where rates appeared to be uniformly low, despite inclusion of urban catchment areas.

The higher rates of psychotic disorders we observed in men,^24^ younger age groups,^5^ and racial/ethnic minorities,^4^ as well as for nonaffective psychoses,^3^ are also frequently reported in the literature. Our study provided further robust evidence of a secondary peak in the risk of psychosis for women older than 45 years, building on previous observations.^30^ Our findings add further evidence to the observation that early intervention services with an upper age limit of 35 years (or lower) may lead to sex-related mental health inequalities^33^: only 50.8% of women (605 of 1190) with psychosis were identified before 35 years of age in our settings, compared with 67.9% of men (1070 of 1577) (Figure 1).

Incidence of FEP varied not only by person, but importantly, by place, suggesting that the social environment may shape incidence patterns of FEP. Our best-fitting models of all FEP and nonaffective psychoses (eTable 3 in the Supplement) suggested that owner-occupancy levels were associated with incidence of FEP, although residual variation at the setting level was not explained by other catchment area–level measures. Acknowledging the potential for reverse causality, owner-occupancy may also be a proxy for a variety of social exposures, most obviously socioeconomic position,^5^ but extending to social stability and cohesiveness, which have previously been associated with psychosis.^26^ The incidence of FEP appeared to be lower in southern Europe, but we found no evidence of variation by latitude in our multivariable models. Nevertheless, settings were located within a narrow band (38°-53° north of the equator), except for Brazil (21° south of the equator). This location may have contributed to our null findings, and the absence of high rates of psychosis in our southern Europe settings, particularly in major urban centers, requires further investigation; incidence patterns with respect to population density in southern European settings appeared to diverge from those observed in northern Europe (eTable 4 in the Supplement).^6^ Variation in the incidence of affective disorders, with lower rates in catchment areas with higher levels of unemployment (eTable 3 in the Supplement), is counterintuitive and unexpected; further research is required to examine this finding.

### Strengths and Limitations

Our findings should be interpreted alongside the strengths and limitations of our study. Our large sample size allowed us to estimate 3 psychotic outcomes in 17 settings with a high degree of precision. To minimize ascertainment bias, all researchers received training via face-to-face epidemiologic training sessions, regular teleconferencing, online training manuals, and interrater reliability protocols. Nonetheless, some limitations of our multinational design need to be acknowledged.

Detection of patients who never present to services is an issue for all epidemiologic studies, and our rate estimates should be interpreted as the treated incidence. Although our overarching case ascertainment method was similar across all settings, some adaptation to local health care systems was necessary. For example, primary care in each catchment area may have referred different proportions of patients with FEP to secondary mental health care services, but referral guidelines were very similar across national settings; these guidelines all urge prompt referral of anyone with FEP. That said, we did not assess whether referral practices were consistent within and between catchment areas. Difference in the average timing of referral may have affected the case mix within the FEP category, but not the overall number of referrals; each center was in a steady state.

Differences in the organization of secondary mental health care services across localities may also have influenced detection of patients. In England and the Netherlands, for example, the widespread commissioning of early intervention in psychosis services may have led to improved detection of new cases of FEP. The leakage study in Brazil revealed a substantial number of new cases at this site (279 [49%]), while similar approaches in 2 French sites (Paris and Val-de-Marne) identified far fewer missed cases (7 [6%] in Paris and 28 [13%] in Val-de-Marne).^30^ Comprehensive, regular contact with mental health services should have helped minimize underascertainment, although some patients, including those treated privately, may have been missed; in general, we believe these biases are unlikely to account for the 8-fold variation between catchment areas.

We used validated semistructured interview schedules, where possible, to obtain standardized research-based OPCRIT diagnoses close to the patient’s first presentation. We have no reason to believe the use of different schedules by setting biased our estimates; indeed, this choice was adapted to local expertise to minimize bias, which may have otherwise arisen from using unfamiliar interview schedules. We considered total incidence of FEP as our primary outcome, as this is useful for, and consistent with, contemporary practice in the management and treatment of psychosis, to allow symptoms to evolve at first presentation and minimize stigma. This practice is also consistent with some evidence of diagnostic instability in the early course of disorder,^34^ particularly for psychotic disorders other than schizophrenia.^35^ Although we relied on clinical diagnoses in a small proportion of patients, this did not alter the interpretation of our findings (eAppendix 2 and eTable 5 in the Supplement).

We classified racial/ethnic minority status as a binary variable, following official definitions used in each country to distinguish racial/ethnic majority and minority groups. This approach may have led to some misclassification, particularly in France, which does not differentiate between people born in mainland France vs its overseas territories, nor is able to identify second-generation (French-born) migrants. This misclassification would have conservatively biased IRRs with respect to racial/ethnic minority status, as would have our decision to code participants with missing data on race/ethnicity (0.2%) to the racial/ethnic majority group.^4^ Our binary race/ethnicity variable may also have permitted residual confounding; risk of psychosis by race/ethnicity will be studied in greater detail in future EU-GEI publications.

We used a consistent method in European catchment areas to estimate measures of the social environment, with comparable data taken from the Brazilian census. European data could only be obtained at the NUTS-2 regional level, which is larger than our catchment areas. Data from this level may have led to exposure misclassification, although the effect of this ecological bias is difficult to determine.

Although we controlled for several risk factors simultaneously (age, sex, racial/ethnic minority status, and catchment area-level factors), we were unable to include other putative risk factors for psychosis, including cannabis use,^36^ urban birth,^6^ family history of psychosis,^37^ childhood trauma,^38^ or genetic risk.^39^ These factors are not routinely available in denominator estimates, but will be investigated in future case-control designs from the EU-GEI study.

## Conclusions

In this international, multicenter study we found that treated incidence of psychotic disorders varied 8-fold between catchment areas after standardization for age, sex, and racial/ethnic minority status. Rates were higher in younger people, men, racial/ethnic minorities, and areas with lower levels of owner-occupied housing, although substantial variation between catchment areas, and by broad diagnosis, remained. These results suggest that there is pronounced variation in the health care burden of psychosis worldwide.

## Notes

Supplement. **eAppendix 1.** Methods **eAppendix 2.** Results **eTable 1.** Recruitment Period and Geographical and Socioeconomic Variables by Catchment Area **eTable 2.** Denominator and Majority Characteristics by Country **eTable 3.** Univariable and Multivariable Random Intercepts Poisson Regression of Nonaffective and Affective Psychotic Disorders **eTable 4.** Effect of Population Density on Incidence of All FEP From Multivariable Random Intercepts Poisson Regression by Country **eTable 5.** Multivariable Random Intercepts Poisson Regression Excluding 367 Participants With Clinically Based Diagnoses **eFigure.** Correlation Between Crude Incidence of all FEP and Geographical and Socioenvironmental Variables
