# Supplementary material for: Treated Incidence of Psychotic Disorders in the Multinational EU-GEI Study
Source: JAMA Psychiatry. 2017 Dec 6;75(1):36–46. doi: 10.1001/jamapsychiatry.2017.3554 (PMC5833538; doi:10.1001/jamapsychiatry.2017.3554)
Supplement: Supplement. — eAppendix 1. Methods eAppendix 2. Results eTable 1. Recruitment Period and Geographical and Socioeconomic Variables by Catchment Area eTable 2. Denominator and Majority Characteristics by Country eTable 3. Univariable and Multivariable Random Intercepts Poisson Regression of Nonaffective and Affective Psychotic Disorders eTable 4. Effect of Population Density on Incidence of All FEP From Multivariable Random Intercepts Poisson Regression by Country eTable 5. Multivariable Random Intercepts Poisson Regression Excluding 367 Participants With Clinically Based Diagnoses eFigure. Correlation Between Crude Incidence of all FEP and Geographical and Socioenvironmental Variables [file jamapsychiatry-75-36-s001.pdf]

Supplementary Online Content

Jongsma HE, Gayer-Anderson C, Lasalvia A, et al; European Network of National Schizophrenia Networks Studying Gene-Environment Interactions Work Package 2 (EU-GEI WP2) Group. Treated incidence of psychotic disorders in the multinational EU-GEI study. *JAMA Psychiatry.* Published online December 6, 2017. doi:10.1001/jamapsychiatry.2017.3554

eAppendix 1. Methods
eAppendix 2. Results
eTable 1. Recruitment Period and Geographical and Socioeconomic Variables by Catchment Area
eTable 2. Denominator and Majority Characteristics by Country
eTable 3. Univariable and Multivariable Random Intercepts Poisson Regression of Nonaffective and Affective Psychotic Disorders
eTable 4. Effect of Population Density on Incidence of All FEP From Multivariable Random Intercepts Poisson Regression by Country
eTable 5. Multivariable Random Intercepts Poisson Regression Excluding 367 Participants With Clinically Based Diagnoses
eFigure. Correlation Between Crude Incidence of all FEP and Geographical and Socioenvironmental Variables

This supplementary material has been provided by the authors to give readers additional information about their work.

© 2017 Jongsma HE et al. *JAMA Psychiatry.*

eAppendix 1. Methods

Classification of ethnicity

Classification of binary majority/minority groups in each setting of the EU-GEI study: In the UK, the Netherlands and Brazil, an ethnicity-based distinction was made between the white British/Dutch/Brazilian majority groups and all minority groups. In Spain and Italy, Spanish-born and Italian-born groups were defined as the majority population, respectively, with all foreign-born groups classified as the minority group. Practically, both definitions led to the identification of a white majority group, since large proportions of adult-aged second- and later-generation groups do not yet exist in Italy or Spain, given substantial immigration is a recent phenomenon. France recognizes all people born in France or its territories as 'French-born', with no further provision for ethnicity, and we followed that definition here.

Deviations from protocol

In Veneto (Italy), data were collected during an earlier time period (2005-7), using a younger upper age-limit (55 years). In Ribeirão Preto (Brazil), Paris and Val-de-Marne (France) we conducted leakage studies to identify potential participants missed during case ascertainment. This involved re-contacting all potential points of contact to screen records to identify potentially missed cases, based on a previous methodology[1]. In Puy-de-Dôme (France), data on minority status was missing for 66% of FEP cases (n=27); this setting was excluded from relevant analyses. In Gouda & Voorhout (the Netherlands), ethical approval was not granted to retrospectively obtain additional clinical information from case notes.

Sensitivity analyses

In sensitivity analyses, we inspected the extent of bias introduced into our results due to diagnoses for a small proportion of cases being from clinical notes rather than OPCRIT. We also tested whether

© 2017 Jongsma HE et al. *JAMA Psychiatry*.

population density was associated with FEP incidence *within* countries in *post hoc* sensitivity analyses, stratifying by country, given the previous literature[2].

© 2017 Jongsma HE et al. *JAMA Psychiatry*.

eAppendix 2. Results

Sample characteristics by diagnostic group

Overall, 78.7% received a non-affective diagnosis (Table 1) (crude incidence: 16.9 per 100,000 person-years, 95%C: 16.2-17.6). A further 19.9% received a diagnosis of affective psychosis (incidence: 4.3 per 100,000 person-years; 95%CI: 3.9-4.6). Remaining participants (1.4%) were diagnosed with psychotic disorder, not otherwise specified. Median age-at-first-contact was younger for non-affective (30 years; IQR: 23-41) than affective psychoses (32 years; IQR: 24-45; Mann-Whitney U-test: -2.5; p=0.01); a higher proportion of women (53.5% vs. 40.5%; χ^2: 30.7; p<0.001) and minority groups (41.1% vs. 36.2%; χ^2: 4.2; p=0.04) were diagnosed with affective psychoses.

Variation in the incidence of non-affective and affective psychoses

The crude and directly standardized incidence of non-affective and affective psychoses varied independently by setting (Table 2). We observed over a 10-fold variation in the crude rate of non-affective psychoses, from 5.2 new cases per 100,000 person-years (95%CI: 3.6-7.5) in Santiago to 57.5 (95%CI: 50.7-65.1) in Southeast London. Crude rates of affective psychoses also varied by setting, from 0.9 per 100,000 person-years in Santiago (95%CI: 0.4-2.1) and Barcelona (95%CI: 0.5-1.8) to 14.9 in Val-de-Marne (95%CI: 11.9-18.6), more than a 17-fold difference. Substantial variation persisted for both sets of disorders after direct standardization for age, sex and minority status (Table 2). Multivariable Poisson regression revealed that, as for all FEP, owner-occupancy was associated with incidence of non-affective psychoses (IRR: 0.76; 95%C: 0.69-0.83). For the affective psychoses, only unemployment was associated with incidence (IRR: 0.30; 95%C: 0.17-0.53) in multivariable regression; elevated rates of both disorders were associated with minority status to a similar extent (eTable 3).

Sensitivity analyses

A small proportion of cases were diagnosed using clinical rather than research diagnoses (N=367; 13.2%), given insufficient data to complete an OPCRIT. More women (14.8% vs. 11.9%; χ^2 on

© 2017 Jongsma HE et al. *JAMA Psychiatry*.

1df=4.4, p=0.03) and participants from minority backgrounds (14.9% vs. 12.2%; χ^2 on 1df=4.4, p=0.04) were diagnosed via clinical ratings, though no differences by age group were observed (χ^2 on 8df=10.2, p=0.25). A higher proportion of affective psychoses were obtained via clinical diagnoses (31.4% vs. 18.5%; χ^2 on 1df=31.7, p<0.001). Excluding people with a clinically-based diagnosis from our analyses did not alter substantially our findings (eTable 4). In *post hoc* multivariable models (eTable 5), population density was positively associated with FEP incidence in England (IRR: 1.17; 95%CI: 1.13-1.21) and the Netherlands (IRR: 1.89; 95%CI: 1.40-2.56), but not Spain or France, while a negative association was observed in Italy (IRR: 0.72; 95%CI: 0.62-0.83).

eReferences

1. Cooper JE, Goodhead D, Craig T, Harris M, Howat J, Korer J. The incidence of schizophrenia in Nottingham. *Br J Psychiatry*. 1987;151(5):619-626. doi:10.1192/bjp.151.5.619.
2. Vassos E, Pedersen CB, Murray RM, Collier DA, Lewis CM. Meta-analysis of the association of urbanicity with schizophrenia. *Schizophr Bull*. 2012;38(6):1118-1123. doi:10.1093/schbul/sbs096.

© 2017 Jongsma HE et al. *JAMA Psychiatry*.

eTable 1. Recruitment Period and Geographical and Socioeconomic Variables by Catchment Area

Setting	Start date	End date	Population density in people/km^2	Latitude	Unemployment (%)	Single household (%)	Owner Occupied (%)	DUP (weeks) (median, IQR)
England								
Southeast London	01/05/2010	01/05/2011	6,162.3	51.5°N	5.3	35.9	35.0	10 (2-50)
Cambridgeshire	01/10/2010	30/09/2013	241.5	52.2°N	3.0	26.7	67.0	9 (3-52)
The Netherlands								
Amsterdam	01/10/2010	01/10/2013	4,908.00	52.4°N	4.0	41.5	46.3	9.5 (2-68)
Gouda & Voorhout	01/12/2010	01/12/2013	4,208.00	52.1°N	4.3	33.3	58.7	4 (1-19)
Spain								
Madrid	23/02/2011	31/12/2012	4,997.2	40.4°N	13.0	23.6	76.8	2.5 (1-7)
Barcelona	20/12/2010	31/12/2012	12,326.5	41.4°N	14.0	23.3	74.3	7.5 (2-52)
Valencia	22/12/2010	31/12/2012	14,467.9	39.5°N	17.8	24.1	82.7	6 (3.5-17)
Oviedo	13/12/2010	31/12/2012	141.9	43.4°N	13.1	27.2	80.0	5.5 (2-32.5)
Santiago	13/12/2010	31/12/2012	102.3	42.9°N	13.8	22.3	77.9	13 (4-79)
Cuenca	08/02/2011	31/12/2012	11.6	40.0°N	17.0	21.6	81.9	26 (2-77)
France								
Paris	01/06/2012	01/06/2014	33,260.0	48.9°N	4.2	35.8	47.6	10.5 (5-25)
Val-de-Marne	01/06/2010	01/06/2014	3,721.2	48.8°N	4.2	35.8	47.6	8.5 (2-71)
Puy-de-Dôme	01/09/2010	31/08/2012	68.5	45.8°N	3.7	36.5	63.9	4 (2-10)
Italy								
Bologna	01/01/2011	31/12/2014	2,744.00	44.5°N	3.2	34.4	71.4	4 (1-15)
Veneto	02/01/2005	31/12/2007	3,100.00	45.4°N	3.1	29.5	76.0	N/A
Palermo	02/10/2010	31/05/2014	4,200.00	38.1°N	8.1	28.5	70.2	3 (1-13)
Brazil								
Ribeirão Preto	01/04/2012	01/04/2015	145.2	21.1°S	4.4	12.4	80.8	13.5 (4-39)

DUP: Duration of untreated psychosis; IQR: interquartile range. N/A: Data from Veneto on DUP were unavailable as incidence data (but not DUP) were collected at an earlier time point: see "Deviations from protocol" in supplementary material

© 2017 Jongsma HE et al. *JAMA Psychiatry*.

eTable 2. Denominator and Majority Characteristics by Country

Country	Denominator source (year)	Denominator type	Ethnic majority	Ethnic minorities
England	Office for National Statistics (2011)	Census	White British	Any other ethnicity
The Netherlands	Statistics Netherlands (2014)	Yearly estimates	Individual and both parents born in the Netherlands	Any other ethnicity
Spain	Instituto Nacional de Estadística (2012)	Yearly estimates	Born in Spain	Born abroad
France	Institute Nationale de la statisique et des études économiques (2011)	Yearly estimates	Born in France and territories*	Born abroad
Italy	L'Instituto nazionale di statistica (yearly)	Yearly estimates	Born in Italy	Born abroad
Brazil	Sistema IBGE de Recuperação Automática (2010)	Yearly estimates	White	Any other skin colour

* French overseas territories are: Guadeloupe, French Guiana, Martinique, Réunion, Mayotte, French Polynesia, Saint Pierre and Miquelon, Wallis and Futuna, Saint Martin, Saint Bartélemy, New Caledonia and French Southern Antarctica.

© 2017 Jongsma HE et al. *JAMA Psychiatry*.

eTable 3. Univariable and Multivariable Random Intercepts Poisson Regression of Nonaffective and Affective Psychotic Disorders

	**Non-affective psychoses**			**Affective psychoses**	
Variable	**Univariable IRR (95% CI)**	**Multivariable IRR (95% CI)**[1]		**Univariable IRR (95% CI)**	**Multivariable IRR (95% CI)**[1]
Individual-level					
Minority status (vs majority)	**1.75 (1.59 – 1.92)**	**1.63 (1.49 – 1.79)**		**1.54 (1.28 – 1.85)**	**1.47 (1.22 – 1.76)**
Setting-level					
Distance from equator (degrees)	1.03 (1.00 – 1.06)[2]	0.99 (0.97 – 1.02)		1.02 (0.97 – 1.08)	1.00 (0.97 – 1.03)
Population density (per 1000 people per km^2)	1.03 (1.00 – 1.07)[3]	1.01 (1.00 – 1.03)[4]		1.00 (0.95 – 1.05)	1.00 (0.96 – 1.03)
Owner-occupancy (10%)	**0.72 (0.65 – 0.80)**	**0.76 (0.69 – 0.83)**		**0.74 (0.58 – 0.93)**	0.95 (0.77 – 1.16)
Single-person households (10%)	1.71 (1.26 – 2.32)	1.10 (0.81 – 1.49)		1.58 (0.93 – 2.68)	0.99 (0.67 – 1.47)
Unemployment (10%)	**0.60 (0.37 – 0.97)**	1.07 (0.79 – 1.47)		**0.27 (0.15 – 0.48)**	**0.30 (0.17 – 0.53)**

IRR: Incidence rate ratio; IRR in bold are statistically significant at *P*<.05

[1]Models adjusted for age, sex, their interaction and, for setting-level variables, ethnicity. IRR for non-significant setting-level variables obtained from a model after additional adjustment for owner-occupancy.
[2]*P*=.07
[3]*P*=.06
[4]*P*=.12

© 2017 Jongsma HE et al. *JAMA Psychiatry*.

eTable 4. Effect of Population Density on Incidence of All FEP From Multivariable Random Intercepts Poisson Regression by Country*

**Country**	**Number of settings**	**Multivariable IRR (95%CI)[1]**	**Wald *P* Value**
England	2	1.17 (1.13-1.21)	<.001
The Netherlands	2	1.89 (1.40-2.56)	<.001
Spain	6	1.01 (0.96-1.06)	.61
France	3	1.01 (1.00-1.03)[2]	.14
Italy	3	0.72 (0.62-0.83)	<.001

IRR: incidence rate ratio
* Brazil excluded from these analyses as only a single setting was part of the study here
[1] Adjusted for age, sex, their interaction, minority status and owner-occupancy
[2] Adjusted for age, sex, their interaction and owner-occupancy only (data on majority status not available for Puy-de-Dôme)

© 2017 Jongsma HE et al. *JAMA Psychiatry*.

eTable 5. Multivariable Random Intercepts Poisson Regression Excluding 367 Participants With Clinically Based Diagnoses

Variable	**All FEP IRR (95% CI)[1]**	**Non-affective psychoses IRR (95% CI)[1]**	**Affective psychoses IRR (95% CI)[1]**
N participants (% full sample)	2,407 (86.8)	1,943 (89.0)	441 (80.1)
Individual-level			
Minority status (vs majority)	**1.55 (1.42 – 1.69)**	**1.57 (1.42 – 1.73)**	**1.38 (1.12 – 1.70)**
Setting-level			
Distance from equator (degrees)	0.99 (0.97 – 1.02)	0.99 (0.96 – 1.02)	1.01 (0.97 – 1.05)
Population density (per 1000 people per km^2)	1.01 (0.99 – 1.03)	1.01 (0.99 – 1.03)	1.00 (0.97 – 1.04)
Owner-occupancy (10%)	**0.75 (0.68 – 0.83)**	**0.75 (0.68 – 0.84)**	0.86 (0.68 – 1.10)
Single-person households (10%)	1.08 (0.77 – 1.52)	1.07 (0.74 – 1.54)	1.20 (0.75 – 1.94)
Unemployment (10%)	0.96 (0.68 – 1.37)	1.10 (0.76 – 1.59)	**0.31 (0.16 – 0.60)**

Legend: Sensitivity analysis to inspect possible bias introduced due to 367 participants diagnoses from clinical diagnoses rather than OPCRIT-based diagnoses

[1]Models adjusted for age, sex, their interaction and, for setting-level variables, ethnicity. IRR for non-significant setting-level variables obtained from a model after additional adjustment for owner-occupancy.

© 2017 Jongsma HE et al. *JAMA Psychiatry*.

eFigure. Correlation Between Crude Incidence Of all FEP and Geographical and Socioenvironmental Variables

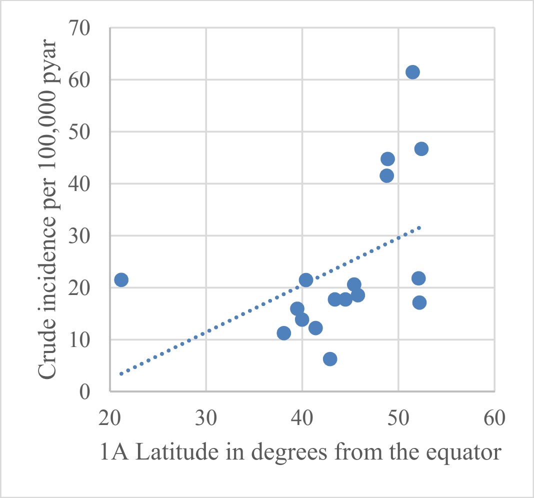

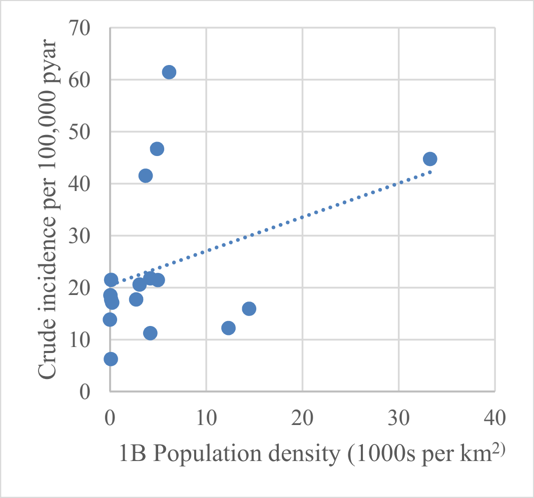

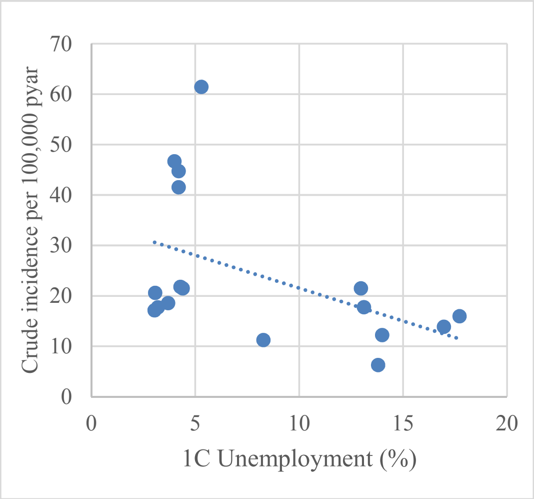

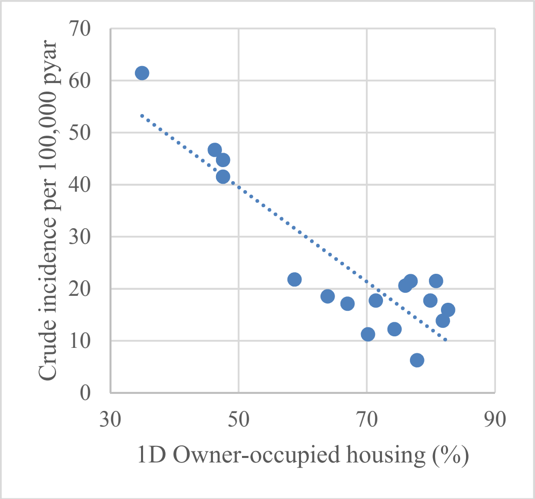

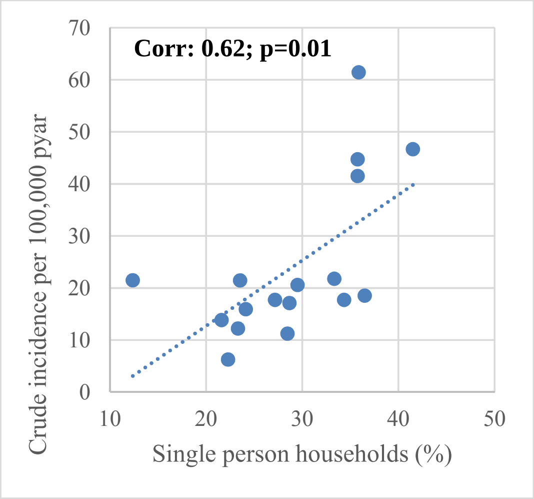

Legend: Figures 1A-1E show scatter plots of the crude incidence of all first episode psychosis and candidate geographical and socioenvironmental variables across 17 catchment areas in the EU-GEI study. Trend-level associations were observed between crude incidence rates and latitude (**1A**; p=0.06) and unemployment rates (**1C**; p=0.06), such that higher latitudes and lower unemployment rates were associated with higher incidence. A striking negative correlation (**1D**; Corr=-0.89; p=0.008) between lower crude incidence rates and higher owner-occupancy rates was also observed in this study.

© 2017 Jongsma HE et al. *JAMA Psychiatry*.
